# The Promise of AI in Detection, Diagnosis, and Epidemiology for Combating COVID-19: Beyond the Hype

**DOI:** 10.3389/frai.2021.652669

**Published:** 2021-05-14

**Authors:** Musa Abdulkareem, Steffen E. Petersen

**Affiliations:** ^1^Barts Heart Centre, Barts Health National Health Service (NHS) Trust, London, United Kingdom; ^2^National Institute for Health Research (NIHR) Barts Biomedical Research Centre, William Harvey Research Institute, Queen Mary University of London, London, United Kingdom; ^3^Health Data Research UK, London, United Kingdom; ^4^The Alan Turing Institute, London, United Kingdom

**Keywords:** artificial intelligence, COVID-19, detection, diagnosis, epidemiology, social control, contact tracing, medical imaging

## Abstract

COVID-19 has created enormous suffering, affecting lives, and causing deaths. The ease with which this type of coronavirus can spread has exposed weaknesses of many healthcare systems around the world. Since its emergence, many governments, research communities, commercial enterprises, and other institutions and stakeholders around the world have been fighting in various ways to curb the spread of the disease. Science and technology have helped in the implementation of policies of many governments that are directed toward mitigating the impacts of the pandemic and in diagnosing and providing care for the disease. Recent technological tools, artificial intelligence (AI) tools in particular, have also been explored to track the spread of the coronavirus, identify patients with high mortality risk and diagnose patients for the disease. In this paper, areas where AI techniques are being used in the detection, diagnosis and epidemiological predictions, forecasting and social control for combating COVID-19 are discussed, highlighting areas of successful applications and underscoring issues that need to be addressed to achieve significant progress in battling COVID-19 and future pandemics. Several AI systems have been developed for diagnosing COVID-19 using medical imaging modalities such as chest CT and X-ray images. These AI systems mainly differ in their choices of the algorithms for image segmentation, classification and disease diagnosis. Other AI-based systems have focused on predicting mortality rate, long-term patient hospitalization and patient outcomes for COVID-19. AI has huge potential in the battle against the COVID-19 pandemic but successful practical deployments of these AI-based tools have so far been limited due to challenges such as limited data accessibility, the need for external evaluation of AI models, the lack of awareness of AI experts of the regulatory landscape governing the deployment of AI tools in healthcare, the need for clinicians and other experts to work with AI experts in a multidisciplinary context and the need to address public concerns over data collection, privacy, and protection. Having a dedicated team with expertise in medical data collection, privacy, access and sharing, using federated learning whereby AI scientists hand over training algorithms to the healthcare institutions to train models locally, and taking full advantage of biomedical data stored in biobanks can alleviate some of problems posed by these challenges. Addressing these challenges will ultimately accelerate the translation of AI research into practical and useful solutions for combating pandemics.

## Introduction

COVID-19, a type of coronavirus disease caused by Severe Acute Respiratory Syndrome Corona-Virus 2 (SARS-CoV-2), has created enormous suffering, affecting lives and causing deaths. The novel nature of the virus means that humans are only newly exposed to the virus (Brüssow, 2020; Wan et al., 2020). First reported in China in December 2019, it was declared by The World Health Organization (WHO) to be a Public Health Emergency of International Concern (PHEIC) on January 30, 2020 and a pandemic on March 11, 2020 (Team, 2020; WHO, 2020). It is an infectious disease that spreads in humans mainly through respiratory droplets produced by an already infected person through sneezing or talking, or airborne transmission (Moriyama et al., 2020). The early symptoms of the disease include persistent high temperature, dry continuous coughing, loss of taste or smell, and difficulty in breathing (Kooraki et al., 2020; Wang et al., 2020a). Severe cases of the disease cause death (Rothan and Byrareddy, 2020; Zhou et al., 2020a).

Due to the ease with which the coronavirus can spread and grow exponentially within the human population, healthcare resources and manpower to rapidly control it is limited as the number of doctors, nurses, and other healthcare workers and resources that could help control it is finite. Moreover, the disease has exposed weaknesses of many healthcare systems around the world. Indeed, the lack of affordable, quick and accurate means of detecting the disease is one of the most important reasons it has rapidly spread (Ai et al., 2020).

Since the emergence of COVID-19, many governments, research communities, commercial enterprises and other institutions and stakeholders around the world have been fighting in various ways to curb the spread of the disease (Chen et al., 2020a; Dong et al., 2020). Science and technology have helped in the implementation of policies of many governments that are directed toward mitigating the impacts of the pandemic and in developing cures and vaccines for the disease. They also offer unique opportunity to support healthcare workers by providing them with tools that would save them time, improve their ability to carry out their job and enhance the management of healthcare systems developed to combat the pandemic, and much more. Many resources have been made available to support the battle against COVID-19, such as datasets (Cheng et al., 2020; Cohen et al., 2020; Zhao et al., 2020a), computing resource (Hack and Papka, 2020), and research funding (Casigliani et al., 2020; Glasziou et al., 2020; Janiaud et al., 2020; Patel et al., 2020; Prudêncio and Costa, 2020; UKCDR, 2020).

The scope of combating COVID-19 using technology is very broad and it includes understanding the socio-economic and medical impacts of the pandemic. From a healthcare perspective, it includes disease detection, diagnosis, and monitoring (Huang et al., 2020a; Kong et al., 2020; Thevarajan et al., 2020; Xu et al., 2020a), epidemiology (Chan et al., 2020; Jin et al., 2020a; Li et al., 2020a), social control (Jin et al., 2020a; Kandel et al., 2020; Qian et al., 2020), virology and pathogenesis (Andersen et al., 2020; Jin et al., 2020a; Lu et al., 2020b; Walls et al., 2020), and drug discovery (Chen et al., 2020b; Phua et al., 2020). For example, during the early phase of the outbreak of the pandemic, China used facial recognition cameras to track infected patients and drones to disinfect public places and broadcast audio messages to the public asking them to stay at home (Ruiz Estrada, 2020). As another example, Taiwan linked its national medical insurance database with the immigration and custom database in order to inform the healthcare practitioners of the travel history of patients (Wang et al., 2020b).

The term artificial intelligence (AI) refers to the study of developing computer algorithms with human-like intelligence to accomplish specific tasks. Machine learning (ML) methods are a set of techniques in AI and includes supervised (Kotsiantis et al., 2007), unsupervised (Barlow, 1989), semi-supervised (Zhu, 2005; Chapelle et al., 2009), and reinforcement learning (Sutton and Barto, 1998). Some of these methods and other terms often encountered in the AI literature are briefly described in Table 1.

**Table 1:** Some terms and methods commonly used in AI.

	**General terms**
Artificial Intelligence (AI)	The concept of developing computer algorithms with human-like intelligence to solve specific tasks.
Deep Learning (or Deep Neural Network)	A set of ML algorithms that are based on neural network (NN) that are used for feature learning. The term “deep” refers to the fact that they have multiple layers between the input and the output layers.
Machine Learning (ML)	A subset of AI and consists of a collection of techniques to achieve AI.
Reinforcement Learning	A set of ML algorithms that is based on the interaction between an agent and its environment. In general, the agent seeks to take actions in the environment by maximizing a cumulative reward.
Supervised Learning	A set of ML algorithms for developing mathematical models using data that consists of both the input and the desired output data.
Unsupervised Learning	A set of ML algorithms for finding underlying structures or patterns in datasets using only the input data.
Convolutional Neural Network (CNN)	A set of DL algorithms that are particularly efficient in developing AI-based applications involving images. CNN acts as the backbone of many well-known neural network architectures (such as U-net) used in image processing.
Random Forests (RF) Method	A set of learning algorithms involving several decision trees and whose output is the class that is the statistical mode (in classification tasks) or statistical mean (in regression tasks) of each of the decision trees. These algorithms are often used for classification tasks and regression analysis problems.
Support Vector Machines (SVM)	A set of supervised learning algorithms that constructs hyperplanes in a high-dimensional space. These algorithms are often used for classification tasks, regression analysis, and other problems. In a classification problem, for instance, out of the many hyperplanes, the one that has the largest distance to the data point of any class is considered the ‘optimal’ classifier.
**Reference List of AI Algorithms Mentioned in this Paper**	
• AlexNet (Russakovsky et al., 2015; Krizhevsky et al., 2017)	
• Artificial Neural Networks (ANN) (Hopfield, 1988; Jain et al., 1996)	
• Adaptive-Network-based Fuzzy Inference System (ANFIS) (Jang, 1993)	
• CNN (LeCun et al., 2015)	
• CNN segmentation model (Region Proposal Network structure) (Ren et al., 2016)	
• CNN model with Inception (Szegedy et al., 2016)	
• Decision Tree (DT) (Breiman et al., 1984)	
• Extreme Gradient Boosting (XGBoost) (Chen and Guestrin, 2016)	
• Generative Adversarial Networks (GANs) (Goodfellow et al., 2014)	
• Gated Recurrent Unit (GRU) recurrent neural network (Cho et al., 2014; Chung et al., 2014)	
• k-mean clustering (Kanungo et al., 2002)	
• k-nearest neighbor (Cover and Hart, 1967)	
• Least Absolute Shrinkage and Selection Operator (LASSO) logistic regression (Tibshirani, 1996)	
• Logistic regression (Hosmer Jr et al., 2013)	
• LSTM (Hochreiter and Schmidhuber, 1997)	
• RF (Breiman, 2001; Liaw and Wiener, 2002)	
• ResNet (He et al., 2016)	
• SVM (Cortes and Vapnik, 1995)	
• U-Net (Ronneberger et al., 2015)	

The applications of AI can be found in many disciplines and industries in modern society, and healthcare is not an exemption. The rapid growth of AI-based techniques and tools in healthcare are addressing complex problems such as identifying previously undiscovered relationships in patient phenotypes (Shivade et al., 2014), optimizing healthcare pathways (Lu and Wang, 2019; Blasiak et al., 2020), and improving accuracy of medical decision making (Bennett and Hauser, 2013; Shortliffe and Sepúlveda, 2018).

Advances and accessibility to high-performance scalable computing equipment have driven the recent popularity of the use of AI in many real-world applications. This development has also prompted an expansion of research into novel AI techniques and algorithms. AI algorithms have the potential to interpret biomedical and healthcare data particularly for tasks where conventional statistical methods are less efficient. The algorithms are even more suitable for datasets of large scale and high dimensions. These algorithms can therefore be used to solve problems such as optimizing care pathways, standardizing clinical diagnosis, identifying relationships in patient phenotypes and developing predictive models (Johnson et al., 2017). While AI-based methods can be used to solve many problems in medicine and healthcare, the success of AI projects, in many cases, depends on the choice of the AI technique, the quality of the dataset to be used and the context associated with the way the dataset is used. For instance, deep learning (DL) algorithms such as convolutional neural networks (CNN) are particularly suitable for computer vision problems such as image segmentation (Shen et al., 2017). Recent advancement in AI research has led to the development of tools in medicine and healthcare that are useful in combating global pandemics. Researchers across several areas of expertise and industries have therefore explored and exploited the use of AI in the battle against COVID-19.

There are many ways in which AI can help in the fight against the COVID-19 pandemic. For example, AI could be used to track the spread of the virus (Al-Qaness et al., 2020; Bandyopadhyay and Dutta, 2020; Carrillo-Larco and Castillo-Cara, 2020; Hu et al., 2020; Jana and Bhaumik, 2020; Huang et al., 2020b; Kavadi et al., 2020; Sameni, 2020), identify patients with high mortality risk (Jiang et al., 2020a; Qi et al., 2020; Xu et al., 2020b; Yan et al., 2020a), diagnose and screen a population for COVID-19 (Ghoshal and Tucker, 2020; Hassanien et al., 2020; Hemdan et al., 2020; Jin et al., 2020b; Maghdid et al., 2020a; Narin et al., 2020; Wang et al., 2020c,e; Wu et al., 2020a; Zhang et al., 2021; Xu et al., 2020c), or reduce the time for diagnosis (Vaishya et al., 2020a). Many of the AI techniques currently being deployed in the battle already existed prior to the pandemic. These techniques include those that can process and understand medical imaging data from modalities such as computed tomography (CT) and X-ray that are being used for detection and diagnosis (Li et al., 2020b; Wang et al., 2020e; Wynants et al., 2020) and those involving non-imaging data that are being used for mortality rate and outcome prediction, prognosis, outbreak prediction, contact tracing and social control of COVID-19 (John and Shaiba, 2019; Bandyopadhyay and Dutta, 2020; Chen et al., 2020e; Goh et al., 2020; Pourhomayoun and Shakibi, 2020; Xu et al., 2020b). Other AI techniques have also found new application areas due to COVID-19. For example, in Shi et al. (2020a), argued for the development of AI-based tools for automated acquisition of medical images in order to optimize the imaging workflow and reduce healthcare practitioners' risk of exposure to the virus by minimizing or eliminating contact with COVID-19 patients.

Several reviews, such as Albahri et al. (2020), Bansal et al. (2020), Bragazzi et al. (2020), Bullock et al. (2020), Jamshidi et al. (2020), Kricka et al. (2020), Kumar et al. (2020), Lalmuanawma et al. (2020), Martin et al. (2020), Naudé (2020), Nguyen (2020), Rasheed et al. (2020), Suri et al. (2020), Shi et al. (2020a), Vaishya et al. (2020b), Zhou et al. (2020b), and Chen et al. (2020c), have been published to showcase the opportunities AI presents in the current effort to fight against COVID-19. In this paper, areas where AI techniques are being used in the detection, diagnosis and epidemiological predictions, forecasting and social control for combating COVID-19 are discussed, highlighting areas of successful applications and underscoring issues that need to be addressed to achieve significant progress in battling COVID-19 and future pandemics. The paper assumes a basic background knowledge of AI techniques, the reader is invited to consult (Raghu and Schmidt, 2020) for further information of these AI methods. Useful introduction to the epidemiology and clinical features of COVID-19 can be found in, for example, C Disease Control (2020).

## AI in COVID-19 Detection and Diagnosis

Detection and diagnosis of COVID-19 is an important part in the fight against the virus. Current diagnostic testing methods are mostly non-invasive methods and they include chest CT and chest X-ray medical imaging, nucleic acid, serologic, and viral throat swab testing methods (Fang et al., 2020; Li et al., 2020c; Lu et al., 2020a; Ozturk et al., 2020; Schwartz, 2020; Zeng et al., 2020). In order to contain the spread of the pandemic and isolate the virus, fast and early detection and tracking of infected patients is crucial and there is clearly the need of innovation in this area (Ai et al., 2020; Fang et al., 2020). In the subsections that follow, AI tools that have been developed for the detection and diagnosis of SARS-CoV-2 and COVID-19 are presented.

### Nucleic Acid Amplification-Based Diagnostics

A type of nucleic acid amplification test (Udugama et al., 2020), the Reverse Transcription-Polymerase Chain Reaction (RT-PCR) test, is one of the most widely used standard testing methods for detecting whether patients have COVID-19 (Ai et al., 2020). The RT-PCR however suffers from inadequate sensitivity, as low as 71% as reported in Fang et al. (2020), as a result of many factors such as low detection efficiency and complicated sample preparation (Lu et al., 2020a; Wu et al., 2020a). This low sensitivity issue results in multiple testing of many patients usually over several days apart in order to obtain a reliable conclusion.

A ML model was reported in Wu et al. (2020a) that uses 11 key blood indices to distinguish between patients with and without COVID-19. The model was developed using the random forest (RF) ML technique and 49 clinical available blood test parameters (consisting of 24 routine hematological and 25 biochemical parameters) from 169 patients with a total number of 253 data samples of which 105 samples are from patients confirmed to have the COVID-19 disease using the RT-PCR test. The remaining samples consisted of 98 samples from patients with common pneumonia and 25 samples each from patients with tuberculosis and lung cancer. The data was divided into 149 training, 33 testing, and 74 validating datasets. The model achieved accuracy of 96.97% on the testing set and 97.95% for the cross-validation set. While this model (which could be further investigated for reliability and also improved further) offers a promising tool for preliminary assessment of suspected patients with COVID-19, it so far has not made it to front-line in the fight against the coronavirus.

### Medical Imaging Diagnostics

Medical imaging is one of the main areas in which AI has found practical applications in medicine and healthcare. Imaging data obtained using different modalities, such as computed tomography (CT), magnetic resonance imaging (MRI) and X-ray, are of high dimension. They contain very rich information that can be used to develop AI applications. Imaging data can be used to generate many useful image-derived phenotypes that are obtained *via* qualitative and quantitative assessment of structural changes (that often characterize the structural and functional properties of an organ), significantly shortening the time for radiologists to accomplish these tasks (Petersen et al., 2017; Suinesiaputra et al., 2018; Mauger et al., 2019). Imaging data can also be combined with non-imaging data from Electronic Health Record (EHR) or elsewhere for identifying biomarkers and predicting disease risk factors (Alaa et al., 2019). Faster and automated reading and interpretation of image workflow can be achieved using AI-based tools (Petersen et al., 2019; Robinson et al., 2019; Bai et al., 2020).

Furthermore, segmentation of medical images is useful as these images are often affected by noise, artifacts, and other uncertainties associated with imaging. Image segmentation involves contouring a medical image into biologically relevant structures, helping to quantify those structures and their functions and to produce measurements that act as biomarkers (such as quantities that can be used to diagnose, monitor, or prognosticate diseases). In particular, AI-based automatic image segmentation tools are beneficial as they help in eliminating variability that would have been introduced if segmentation were manually done. Consequently, the use of these AI-based technologies has contributed in the fight against COVID-19 (Bullock et al., 2020). Medical imaging modalities, such as chest CT and X-ray imaging, have provided significant support to clinicians in diagnosing COVID-19 (Apostolopoulos and Mpesiana, 2020; Bernheim et al., 2020; Kanne, 2020). A typical workflow for diagnosing COVID-19 with medical imaging modalities involves the following three phases: (i) pre-scan preparation according to a given protocol; (ii) image acquisition; and (iii) diagnosis.

AI tools for COVID-19 diagnosis with medical images often consist of one or a combination of several AI models (or networks) involving the following two main components: (i) image segmentation models, and (ii) image classification. Image segmentation is used to mark and identify the region of interest (ROI) while an image classification task extracts features from the ROI and uses those features as a basis for classifying (diagnosing) the images.

#### CT Medical Imaging

Chest CT images are being used for early diagnosis of COVID-19 by identifying ground-glass opacity (GGO) around the subpleural region (Ai et al., 2020; Chung et al., 2020; Fang et al., 2020; Kanne, 2020; Wong et al., 2020). In Pan et al. (2020), the dynamic radiological patterns in chest CT images of COVID-19 patients was reported with the following four stages identified: (i) 0–4 days: early stage; (ii) 5–8 days: progressive stage; (iii) 9–13 days: peak stage; and (iv) 14 days and beyond: absorption stage. These distinct manifestations of COVID-19 in CT images provide evidence and severity of the disease that are exploited using AI systems for diagnosing the disease. Generally, the process of COVID-19 diagnosis with CT images involves the following steps: (i) image pre-processing, (ii) image segmentation, (iii) classification, and (iv) model evaluation.

AI tools for COVID-19 diagnosis with CT images involving lung tissue segmentation are reported in Jin et al. (2020b), Li et al. (2020b), and Xu et al. (2020c). As an example, the AI system presented in Jin et al. (2020b) classifies chest CT input image slices into the following four categories: non-pneumonia, non-viral community acquired pneumonia (CAP), influenza-A/B and COVID-19. The predicted class of an image is that which has the highest probability among the four classes. This AI system was developed using two main DL algorithms: the U-Net for performing the lung segmentation task and the ResNet for performing the classification (diagnosis) task. The ROI in the image include the lung, lung lobes, bronchopulmonary segments, and infected lesions. The dataset consists of 10,250 CT scans from three centers in China and three publicly available external databases. This multi-center dataset was from 7,917 subjects consisting of 3,686 scans of COVID-19, 2,886 scans of CAP, 132 scans of influenza-A/B and 3,546 scans of non-pneumonia subjects. The COVID-19 subjects were all confirmed using the RT-PCR diagnostic test. The imaging dataset of 10,250 was divided into a total training dataset of 5,104 and a total testing dataset of 5,146. As a measure of accuracy, on internal testing dataset of 3,203 images (out of the 5,146) the AI system achieved an AUC of 97.17%, a sensitivity of 90.19% and a specificity of 95.76%. It achieved an AUC of 97.77% on the remaining (external) dataset of 1,943 images.

As another example, the AI system presented in Xu et al. (2020c) classifies chest CT input image slices into the following three categories: influenza-A viral pneumonia (IAVP), COVID-19 and irrelevant to infection (i.e., cases that do not belong to the other two categories) cases. The predicted class of an image is that which has the highest probability that the image belongs to it. This AI system consists of two main DL algorithms: a three-dimensional (3D) CNN segmentation model (Region Proposal Network structure) for performing a lung segmentation task and a ResNet-based model for performing the image classification task. The dataset consists of 618 CT scans from three hospitals in China's Zhejiang Province of which 110 subjects (219 scans) were confirmed of COVID-19 using the RT-PCR diagnostic test; 224 subjects (224 scans) had IAVP, and the remaining 175 scans are healthy subjects. The imaging dataset of 528 (189 COVID-19 cases plus 194 IAVP cases plus 145 healthy cases) were used for training and validation, and the remaining 90 scans (30 COVID-19 cases plus 30 IAVP cases plus 30 healthy cases) were used as a testing dataset. On the testing dataset, the AI system achieved an f_1_-score of 83.9% for COVID-19 cases, 84.7% for IAVP cases, 91.5% for healthy cases and an overall accuracy rate of 86.7%.

Several AI systems, such as Ardakani et al. (2020), Chen et al. (2020d), Gozes et al. (2020), Kang et al. (2020), Li et al. (2020b), Shi et al. (2020b), Song et al. (2020), Tang et al. (2020) and Wang et al. (2020c,d), have been developed for diagnosing COVID-19. Compared to the examples in the two preceding paragraphs, these AI systems mainly differ in their choices of the algorithm for image segmentation of the ROI and the algorithm used for classification or diagnosis. The image segmentation algorithms used include U-Net, U-Net++, V-Net, and others, and the image classification algorithms include ResNet and CNN model with Inception.

In order to address the problem of lack of large datasets of COVID-19 patients for developing AI-based models, researchers, such as in Jin et al. (2020b) and Zhao et al. (2020a), have used different techniques such as data augmentation and transfer learning, to solve the CT image classification problems for COVID-19 diagnosis. In Qian et al. (2020), the classification task was to classify COVID-19 patients into those that will have short-term and long-term hospital stay. Some AI models, such as Shi et al. (2020b,c), went further after the image segmentation task to predict the severity of COVID-19 in patients using algorithms such as least absolute shrinkage and selection operator (LASSO) logistic regression model and RF.

#### X-Ray Medical Imaging

The X-ray technology is a very popular imaging modality in medical imaging (Wang et al., 2017). The CT and X-ray medical imaging modalities have been more widely accessible and used to provide evidence and for COVID-19 diagnosis compared to other imaging modalities due to their fast acquisition. In fact, in many healthcare centers and hospitals, X-ray imaging, due to its accessibility and quickness to obtain, is often the first-line imaging modality for suspected COVID-19 patients (Bullock et al., 2020; Shi et al., 2020a). Although the chest X-ray images are less informative compared to CT images for diagnosing COVID-19 due to lower sensitivity of chest X-ray images, the popularity and availability of X-ray imaging facilities means that it is widely used for the diagnosis of the disease. As with chest CT imaging, chest X-ray imaging is being used for diagnosis of COVID-19 by identifying ground-glass opacity (GGO) around the subpleural region, and these manifestations of COVID-19 in chest X-ray images provide evidence and classification of severity of the disease that are being exploited using AI systems for diagnosing the disease.

In general, the process of COVID-19 diagnosis with chest X-ray images using AI tools involves the following steps: (i) image pre-processing, (ii) image classification, and (iii) model evaluation. In other words, compared to the AI tool for CT images, the image segmentation process is absent although some researchers, such as Hassanien et al. (2020), included classical computer vision methods (i.e., not AI-based methods, such as image thresholding) for carrying out the image segmentation step as well. The AI-based image segmentation part of the process is particularly difficult in the case of chest X-ray images given that the ribs are projected onto other tissues on these images (Chen et al., 2020c) so researchers often skip that step completely. Classification tasks were binary, multi-class, multi-labeled or hierarchical classifications (Albahri et al., 2020).

Several AI systems, such as Ghoshal and Tucker (2020), Hassanien et al. (2020), Hemdan et al. (2020), Maghdid et al. (2020a), Narin et al. (2020), Wang et al. (2019, 2020d), Zhang et al. (2021), have been developed for diagnosing COVID-19 using chest X-ray images. These AI systems mainly differ in their choice of the algorithms used for the image classification task and often combine several algorithms (often, to achieve a feature extraction step before a classification process). The image classification algorithms that are being used include Support Vector Machines (SVM), CNN, AlexNet, ResNet, and CNN model with Inception.

The large number of AI techniques available for diagnosing and classifying a disease means that it can be daunting to select the most appropriate technique (in terms of accuracy and computation efficiency) for a given problem given that many of the researchers have used different (and sometimes conflicting) evaluation criteria for their adopted techniques (Alsalem et al., 2018, 2019; Zaidan et al., 2020). In Albahri et al. (2020), carried out a literature review of AI techniques involving medical images that are being used for diagnosing COVID-19 in an attempt to evaluate and establish benchmarking procedures for these techniques. A detailed description of the proposed methodology for the evaluation and benchmarking of these AI techniques is beyond the scope of this paper and the reader is invited to consult (Albahri et al., 2020) for further information.

### Other Tools for Diagnostics

In Schuller et al. (2020), presented a potential computer audition tool that uses AI-based speech and sound analysis to COVID-19 diagnosis. The authors surveyed automatic recognition and monitoring of contextually significant phenomena from speech or sound, such as dry and wet coughing or sneezing sounds, pain, speech under cold, and breathing for diagnostic exploitation using AI techniques such as Generative Adversarial Networks (GANs) (Pascual et al., 2017).

In Wang et al. (2020f), an AI-based classification model was proposed that is able to distinguish respiratory pattern from six other viral infection respiratory patterns using the Gated Recurrent Unit (GRU) recurrent neural network algorithm with bi-directional attention mechanism. As measures of accuracy, the reported precision, recall, f_1_-score, and accuracy of the model were 94.4, 95.1, 94.8, and 94.5%, respectively. Other models that use respiratory or coughing data for COVID-19 diagnosis can be found in Brown et al. (2020), Imran et al. (2020), and Jiang et al. (2020b).

Researchers, such as in Maghdid et al. (2020b), have also proposed frameworks for using in-built mobile phone sensors including cameras (to scan CT images, for example), temperature sensors, and so on, for COVID-19 diagnosis. The computer audition tools for diagnosing COVID-19, models that use respiratory or coughing data for COVID-19 diagnosis and other AI-based computational frameworks that use speech and sound analysis and in-built mobile sensors, such as Iqbal and Faiz (2020) have not yet gone beyond the conceptual phase.

## AI in Epidemiology

In the subsections that follow, AI tools that have been developed for epidemiological predictions, forecasting and social control for combating COVID-19 are presented.

### AI for Prognosis

The ability to forecast possible patient outcomes is vital in the planning and management of a pandemic such as COVID-19. In order to improve prognosis and not to overwhelm healthcare systems, the ability to predict number of patients at risk of developing acute respiratory distress syndrome and patients at risk of hospitalization or death can be very important (Bullock et al., 2020). In the fight against MERS Co-V, for example, AI-based models have been used to predict prognosis in patients' infection (in particular, patients' recovery) using patients' profession (e.g., whether healthcare workers or not), age, pre-existing healthcare conditions, and disease severity as model input parameters (John and Shaiba, 2019). Similar AI-based applications and methods have been developed for Ebola patients (Colubri et al., 2016; Riad et al., 2019). These and other similar tools can help, for example, to assess healthcare preparedness for a pandemic and to determine treatment methods and resource allocation during a pandemic, and some of the these algorithms could be adapted for decision making in the management of COVID-19 (Bansal et al., 2020).

Epidemiological research is a vast area, and a huge amount of publications on epidemiological modeling of COVID-19 using well-established classical methods have surfaced since the beginning of the pandemic (Cooper et al., 2020; Jewell et al., 2020; Ndairou et al., 2020). Recently, researchers have proposed several AI-based techniques for predicting mortality rate, long-term patient hospitalization (Qi et al., 2020) and patient outcomes for COVID-19 (Jiang et al., 2020a; Yan et al., 2020a). AI-based techniques that have been used to accomplish the prediction tasks include artificial neural networks (ANN), SVM, and XGBoost. For example, in Pourhomayoun and Shakibi (2020), using dataset of more than 117,000 confirmed COVID-19 patients from 76 countries described in Xu et al. (2020b), the authors used several AI-methods (including SVM, ANN, RF, Decision Tree (DT), logistic regression, and k-nearest neighbor) for the prediction of mortality rate of COVID-19 patients using 112 features consisting of 80 features from patients' doctors notes and health status and 32 features from patients' demographic and physiological data.

### AI for Outbreak Forecasting and Control

The development of forecasting models in order to help policy makers and other stakeholders understand the progression of the pandemic is one of the first areas where mathematical methods were applied to tackle the COVID-19 pandemic. It is therefore not surprising that outbreak forecasting is also one of the first areas in which AI methods have been applied in the fight against the COVID-19 pandemic (Rasheed et al., 2020). There are many existing statistical and dynamic methods for modeling the spread of infectious diseases and understand the impact of interventions to curb these diseases, such as mass vaccination or social distancing, in any given population (Anderson and May, 1979; May and Anderson, 1979; Mena-Lorcat and Hethcote, 1992; Isham and Medley, 1996; Vynnycky and White, 2010; Siettos and Russo, 2013; Pastor-Satorras et al., 2015). Several of these methods have been used to understand and forecast the spread of COVID-19 from available data (Karako et al., 2020; Sameni, 2020; Wu et al., 2020b; Zhao et al., 2020b). These methods can be used to determine transmission factors in order to establish preventive and control measures for the pandemic.

The majority of AI applications developed in the fight against COVID-19 have focused on predicting national and local statistics such as the number of confirmed cases, deaths, and people recovered from COVID-19 (Bullock et al., 2020). AI models that have been developed for outbreak predictions include (Al-Qaness et al., 2020; Bandyopadhyay and Dutta, 2020; Carrillo-Larco and Castillo-Cara, 2020; Hu et al., 2020; Jana and Bhaumik, 2020; Huang et al., 2020b; Kavadi et al., 2020; Sameni, 2020), and the modeling techniques used for these models include CNN, long short-term memory (LSTM), adaptive-network-based fuzzy inference system (ANFIS), partial derivative regression and non-linear machine learning (PDR-NML) (Kavadi et al., 2020), SVM and k-mean clustering.

For example, in Carrillo-Larco and Castillo-Cara (2020), a model based on the k-means clustering algorithm was developed and used to categorize countries based on the number of confirmed COVID-19 cases using a dataset that contains features such as the prevalence of HIV/AIDS, diabetes, and tuberculosis in 156 countries in addition to data on the number of COVID-19 related deaths, confirmed cases and recovered cases. In Al-Qaness et al. (2020), an ANFIS-based model was developed to estimate and forecast the number of confirmed cases of COVID-19 10 days ahead using data of previously confirmed cases. And in Ribeiro et al. (2020), for 10 Brazilian states with a high daily COVID-19 incidence, a stacked ensemble of learning algorithms [autoregressive integrated moving average (ARIMA), cubist regression (CUBIST), RF, ridge regression (RIDGE), SVM] with a Gaussian process (GP) meta-learner was used to conduct 1, 3, and 6-days ahead time series forecasting of the COVID-19 cumulative confirmed cases, achieving errors in a range of 0.87–3.51%, 1.02–5.63%, and 0.95–6.90% in 1, 3, and 6-days-ahead predictions, respectively.

In addition, some of these AI-based models, such as in Kavadi et al. (2020), have reported prediction accuracies that are superior to traditional linear regression-based methods. Researchers, such as in Fong et al. (2020), have also proposed techniques for comparing these different models that have mostly been developed using different architectures and trained with non-identical hyperparameters.

### AI for Contact Tracing and Social Control

The implementation of indiscriminate lockdowns in several countries in an attempt to control the COVID-19 pandemic have had severe social and economic consequences. Despite the physical distancing measures in-place when some of the lockdown restrictions where gradually relaxed, other public health measures were necessary in order to control the pandemic (Hellewell et al., 2020; Hope et al., 2020; Park et al., 2020; Salathé et al., 2020; Kretzschmar et al., 2020a,b), and contact tracing (whether conventional methods that rely on interviewing COVID-19 patients or mobile phone application technology) has been one of the methods that have been adopted in many parts of the world for this purpose. Contact tracing involves contact identification, contact listing and contact follow-up (Kricka et al., 2020).

For contact tracing purposes, mobile applications that have been deployed to notify every participating user that a person with COVID-19 was within a certain distance of the user for more than a specific amount of time include COVIDSafe (Australia), Ketju (Finland), CoronaApp (Germany), StopCovid (France), NZ COVID Tracer (New Zealand), TraceTogether (Singapore), NHS Covid-19 App (United Kingdom), to mention a few (Lalmuanawma et al., 2020). As far as we know, none of these digital technologies have been confirmed to use AI-based models as tools, for example, in identifying those in contact with a COVID-19 patient [in Lalmuanawma et al. (2020) though, there is a report that AI tools are being used but this could not be confirmed in the references provided by the authors]. There are however promises [see Kricka et al. (2020), for example] that data gathered through these applications could be exploited for developing AI-based tools in the future.

In addition, AI techniques have been used to develop applications for managing and control the spread of the COVID-19 pandemic. Technologies, such as drones and surveillance cameras equipped with AI-based models for enforcing social isolation (Ahmed et al., 2021), have been reported. As for the impacts of the various social control strategies, the reader is invited to consult (Chang et al., 2020; Hellewell et al., 2020; Kissler et al., 2020; Koo et al., 2020) for further information.

## Discussion

Promising and encouraging AI-based techniques and frameworks for the detection, diagnosis, and epidemiological predictions, forecasting and social control of COVID-19 have been proposed in the fight against the disease. For these AI techniques to gain wide acceptance and use in practical clinical settings however, there would need to be a framework on how these models would be incorporated into clinical practice systems. Importantly, the models, which have been developed with mostly limited amount of data using different algorithms and architectures, would need to be trained and validated with larger amount of data and issues such as overfitting and biasness should be appropriately addressed. Evaluating and comparing the performance of AI models is crucial but challenging. This is partly due to complex relationships amongst the choice of algorithms, architectures, hyperparameters, and the quality and amount of data used for these models.

In addition, many (if not the majority) of the proposed or developed AI-based techniques and models for COVID-19 diagnosis and epidemiological forecasting have not been externally evaluated and caution must be exercised in the interpretation of these results. Indeed, despite the urgency for the publication of research results during the COVID-19 pandemic, these models cannot be used in clinical practice in their current form as critical review and external assessment of the techniques and models with multi-center datasets should be carried out.

To illustrate the scale of the lack of external evaluation problem with an example, consider a recent study presented in Yan et al. (2020b) where the authors have used blood samples from 485 infected patients in the region of Wuhan, China, to identify crucial predictive biomarkers of disease mortality using AI-based tools. In this relatively simple severity and outcome prediction task, and with a small validation sample size and no external model evaluation, the authors have used the XGBoost classifier method to identify three biomarkers [namely, lactic dehydrogenase (LDH), lymphocyte count and high-sensitivity C-reactive protein (hs-CRP)] that will allow the prediction of the mortality of COVID-19 patients more than 10 days in advance with reportedly more than 90% accuracy. External evaluation of this result by several other researchers, such as in Barish et al. (2020), Giacobbe (2020), Quanjel et al. (2020), and Dupuis et al. (2021), has shown that the results of Yan et al. (2020b) have limited clinical utility as it was impossible to replicate the findings and arrive at the same conclusion. If a huge external evaluation problem exists even for simpler problems (such as prediction and forecasting problems), one can only imagine the scale of the problem when using AI-based model for more complicated problems such as those involving images (computer vision-related problems).

AI has huge potential in the battle against the COVID-19 pandemic. Despite several AI approaches and techniques proposed for the detection, diagnosis and epidemiological predictions, forecasting, and social control in the combat against the pandemic, successful practical deployments of these AI-based tools have so far been limited. There are challenges that have led to the limited applicability of these AI-based tools. In the following paragraphs, some of these challenges are discussed with some suggestions of how some of these obstacles may be tackled in order to achieve significant progress in battling COVID-19 and future pandemics using AI techniques.

### Data Accessibility

One of the key challenges that AI experts have faced during the COVID-19 pandemic is the lack of access to sufficiently large datasets for training and external validation of AI models upon which deployable and successful applications depend. In order to tackle this problem for COVID-19 and future pandemics, healthcare centers would need a dedicated team with expertise in medical data collection, privacy, access, and sharing. In short, data governance frameworks and protocols for pandemics and other emergency times will need to be designed and put in place.

One of the sources of data that has not been taken full advantage of so far for developing AI-based applications and solutions during the COVID-19 pandemic are data from biobanks. Biobanks provide infrastructure for the collection and storage of biomedical data, including data related to health records and lifestyle of participants, with the aim of advancing scientific research and improving healthcare. They are often large databases that can store imaging data, text data from electronic health record (EHR) and lifestyle information, and numerical data obtained by physical measurements of consented participants. Several types of biobanks exist around the world with different population sizes, including genetic banks, blood banks, and tissue banks. These biobanks contain valuable data that can provide insights into how the health of a population develops over years and provide a rich source of data that can be harnessed to unveil complex relationships amongst variables [such as environmental (Wright et al., 2002; Hall et al., 2014), lifestyle choice (Rutten-Jacobs et al., 2018; Said et al., 2018), and genetics (Arnau-Soler et al., 2019; Wang et al., 2019)] that are associated with COVID-19. Biobanking is particularly useful in that it provides a unified data repository with mostly standardized data collecting protocols. In contrast, the hospital data are “messy” due to the nature of data collection and storage across multiple repositories. Examples of biobanks include the Kaiser Permanente's Research Program on Genes, Environment and Health (RPGEH) with 200,000 participants (Kaiser Permanente, 2020), the UK Biobank with 500,000 participants (Biobank, 2014), China Kadoorie Biobank with 500,000 participants (Chen et al., 2005a, 2011), India's Chennai biobank with 500,000 participants (Gajalakshmi et al., 2007), and Biobank of Vanderbilt University Medical Center (BioVU) with over 1.4 million participants (Roden et al., 2008). Not all these biobanks have data of COVID-19 patients. The UK Biobank, one of the largest biobanks in the world in terms of data volume and depth including multi-organ imaging, is an example of one that has been integrated with pre-existing data of COVID-19 patients. UK Biobank's data has been used for research related to COVID-19 [for example, see Armstrong et al. (2020), Atkins et al. (2020), Grant and McDonnell (2020), Hastie et al. (2020), Jimenez-Solem et al. (2020), Kenneth and So (2020), Pereira et al. (2020), Sattar et al. (2020), Toh and Brody (2020), and Zimmerman and Kalra (2020)]. Few AI-based applications, such as in Jimenez-Solem et al. (2020), Kenneth and So (2020), Pereira et al. (2020), Toh and Brody (2020), and Zimmerman and Kalra (2020), exist that have used biobanks' data for their development, and it is likely that the use of biobanks' data for the development of AI solutions will increase in the near future.

In addition, researchers, such as in Brisimi et al. (2018), Lee et al. (2018), Rieke et al. (2020), Li et al. (2020d), and Xu et al. (2020d), have proposed the use of federated learning (FL) whereby, rather than participating healthcare institutions hand over healthcare data to AI experts to develop AI models, AI experts will handover training algorithms to the healthcare institutions to train their models locally. The AI experts only get the model or model parameters in return—thus, eliminating some of the problems of data governance and privacy associated with data transfer between different parties while giving access to large amount of data. FL is not without its challenges, such as lesser accuracy of the final model (Li et al., 2020d), and the reader is invited to consult (Brisimi et al., 2018; Lee et al., 2018; Li et al., 2020d; Xu et al., 2020d) for further information of this approach.

### External Evaluation

**Figure 1:**
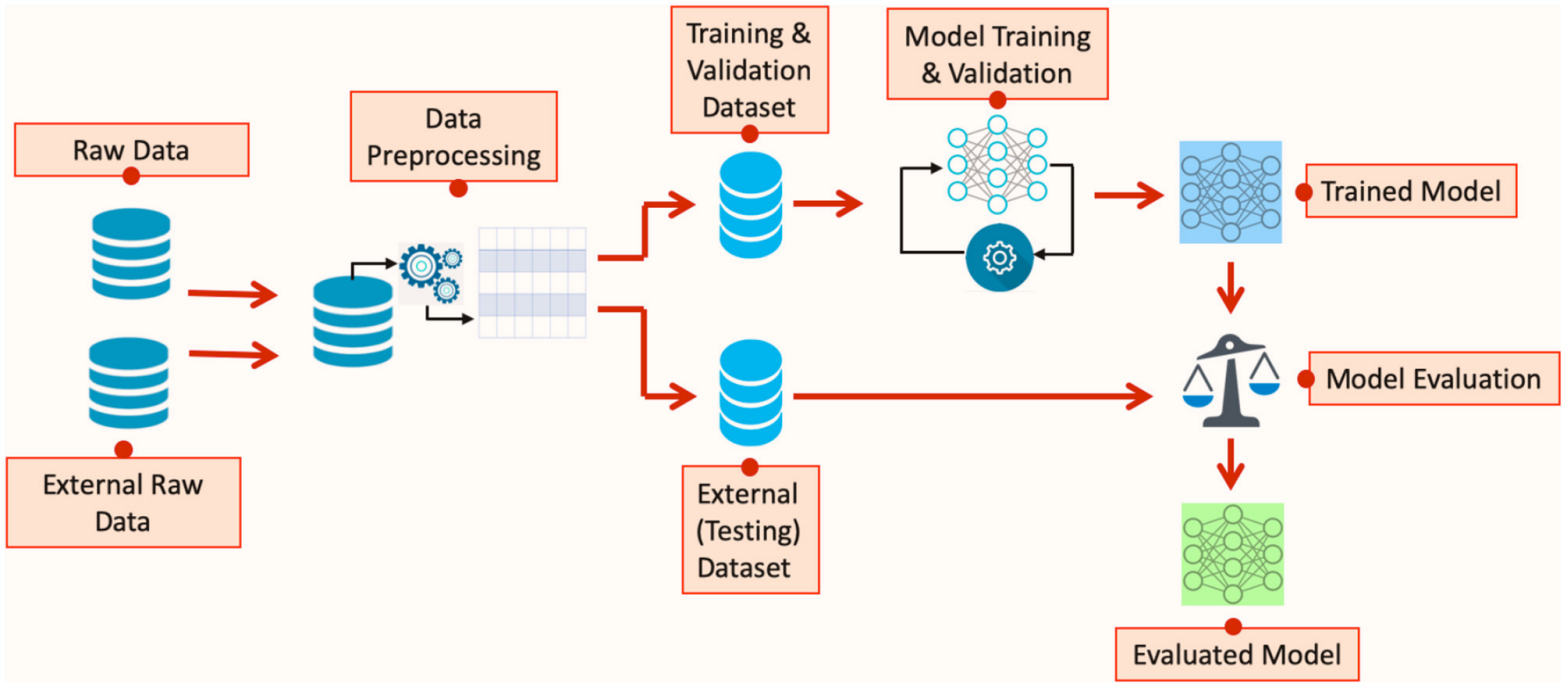
Steps in developing models using AI algorithms.

Many of the developed AI-based techniques and models for COVID-19 diagnosis and epidemiological forecasting have not been externally evaluated. External model evaluation helps in assessing the generalisability of the predictions on independent datasets and ensures that the model has learnt the underlying features of the process that produces the data rather than “memorized” the features of a particular set of data. For illustration, Figure 1 shows the steps in developing models using AI algorithms, highlighting the model evaluation stage of the development process.

Many publicly available datasets for COVID-19 diagnosis do not necessarily generalize to the whole population (i.e., they are usually for a specific country or regions of a country or a specific number of hospitals). The implication is that most ML models based on them will be biased (He and Garcia, 2009), which can reduce the performance of the models in practical settings (Chawla et al., 2002) and can promote healthcare inequalities (Petersen et al., 2019). Many mainstream ML algorithms for classification problems, including SVM, decision trees, and nearest neighbor, were developed based on the assumption that the dataset has balanced class distribution (Chen et al., 2005b; Almogahed and Kakadiaris, 2015), resulting in significant error when classifying the minority class. Algorithms that have been developed to overcome this problem algorithmically or at data level can be found in Hart (1968), Kubat and Matwin (1997), Laurikkala (2001), Barandela et al. (2003), Oh (2011), and Almogahed and Kakadiaris (2015). In addition, it is important for published research to report the pre-processing, the cleaning and the feature engineering steps applied to the data used for developing AI-based solutions.

### AI Regulatory Landscape

Recently, frameworks for strong regulatory and ethical requirements of AI-based clinical utility tools are being developed but significant hurdles still persist (Petersen et al., 2019). Many AI experts are unaware of the regulatory landscape governing the development of AI tools in healthcare and have not considered this matter in their development. Proof of model performance is not sufficient. Issues, such as model biasness, safety, effectiveness, and benefit-versus-harm analysis have mostly been ignored by developers of many critical AI-based healthcare technologies.

### Collaboration Between AI Experts and Clinicians

While AI provides the opportunity to reduce the time for disease diagnosis and improve accuracy, the workload of healthcare professionals is very high during a pandemic. The impact of this includes the difficulty for healthcare professionals to be up to date with the progress being made in areas relevant to their work. It has also hindered their contribution toward that progress. The absence and lack of engagement of clinicians to contribute and review research results during the COVID-19 pandemic has contributed to the limited impact, reliability and clinical utility of many of these research findings. The COVID-19 pandemic has highlighted the importance of domain specific knowledge in AI. It is not sufficient for clinicians to handover data to AI experts who understand how to develop and use classical AI algorithms. Rather, it is important for the clinicians to work with AI experts to help them understand the context of the solutions being developed, to help them interpret the results from those solutions, and to guide them on how those solutions could be used and integrated into existing clinical healthcare pathways or workflows. Thus, multidisciplinary research collaborations will no doubt accelerate the translation of AI research into practical solutions in healthcare and funding bodies could help in this by ensuring multidisciplinary collaborations as a condition for funding. An important lesson that should be learnt in using AI techniques for combating COVID-19 and future pandemics is that the applicability of these techniques is limited if AI experts work in isolation. Important progress in healthcare using AI technologies can be achieved only in a multi-disciplinary setting where clinicians, epidemiologists, computer scientists, software developers, AI experts and others work together to achieve the common goal of improving healthcare services through innovative technologies.

### Public Engagements Over Privacy Concerns

While AI-based technologies embedded in digital systems have played a role in controlling the spread of COVID-19 and the general management of the disease by many governments across the world, the concerns of the general public over privacy have had an impact on the acceptance of many of these technologies and even other potential applications. Consider, for instance, the contract tracing mobile applications that many governments have deployed as a tool for controlling COVID-19, concerns over the possibility that data gathered through these applications could be exploited for other purposes has meant that the general public have been very reluctant in using them (Clark et al., 2020; Lewis, 2020). It has also meant that tools applicable to one country (such as China's use of facial recognition cameras to track infected patients or the linking of the national medical insurance database with the immigration and custom database in order to inform the healthcare practitioners in Taiwan of the travel history of patients) may not be applicable to others.

A framework that will ensure transparency over the legal basis of data use, that data collection is safe and that there are controls and mechanisms to protect misuse of data is critical now and in future. Thus, while it is essential to gather data to address the challenges posed by a pandemic, the authorities would need to do work on gaining the trust of the population through effective engagements with all stakeholders on the mechanisms that would be in place in order to protect privacy and data misuse.

### Potential Misuse of AI Applications

One of the dangers of reliance on AI applications during a pandemic is the potential for misuse. Medical imaging involves several stages including image acquisition, reconstruction, and transmission for storage using Digital Imaging and Communications in Medicine (DICOM) protocol. A cyber-attack could disrupt the use of the devices such as CT devices that can be critical for disease diagnosis during a pandemic (Mahler et al., 2018). With the advent of advanced AI techniques such as generative adversarial network in medical imaging (Yi et al., 2019), one can envisage sophisticated scenarios where AI technologies are used for cyber-attacks that can alter the output of imaging modalities (for instance, by removing or adding a tissue to medical images) altering the results of medical examination, which could lead to fatal consequences. With increasing cyber-attack activities during COVID-19 (Lallie et al., 2021; Muthuppalaniappan and Stevenson, 2021), healthcare providers must be prepared for preventing the occurrence and also detecting and mitigating the impacts that these type of AI attacks will cause when they occur.

In addition, while FL can resolve data governance issues, it does not necessarily guarantee data security on its own as it may be possible to reconstruct parts of the training dataset from the weights on decentralized computer nodes (Kaissis et al., 2020). This possibility can allow attackers to steal sensitive personal information in the training datasets from the nodes and even reconstruct medical images with high degree of accuracy (Fredrikson et al., 2015; Hitaj et al., 2017), leading to patient confidentiality violations.

Problems associated with data imbalance, variability and incompleteness resulting from the use of datasets that are not accurate representation of the population on which AI models was built for can lead to biased treatment of certain ethnic, sex, age, and other groups. In many cases, these data biases are often introduced inadvertently by AI algorithm developers but unscrupulous individuals can take advantage of this to exacerbate bias from cultural prejudices and increase disparities in delivering healthcare services. Moreover, misuse of AI models can also result when the datasets used for model training do not take into account future use-case conditions; for example, radiologists can easily adapt to change in MRI field strength and breathing motion artifacts but these changes will affect the performance of AI models unless they have been specifically allowed for during the training of the models (Brady and Neri, 2020).

These issues of misuse of AI as highlighted here show that it is important to provide safeguards to ensure that new AI solutions during a pandemic are assessed before being deployed at scale. It is important to emphasize that these challenges posed by AI are not necessarily associated with the limitations of AI *per se* (Rodriguez et al., 2018). Rather, they apply to particular use-cases and emphasize the importance of understanding the relationships that AI models use in arriving at their predictions. As such, guards against spurious predictions must be put in place in order to limit data misuse.

We finish by noting that, recently, there have been several promising initiatives from key players (e.g., government bodies, commercial institutions, and policy makers) to collect and manage data in order to address or alleviate some of the problems highlighted in this paper. We mention a few of them in the following paragraphs.

In the United Kingdom, NHSX, the government's unit responsible for developing and setting national policy on digital, data and technology for National Health Service (NHS), has developed the National COVID-19 Chest Imaging Database (NCCID) in order to collect patient data and facilitate research and the development and validation of technologies that are promising for improving COVID-19 care (NHSX, 2021). The categories of collected data include chest X-ray, CT, and MR images including those performed in the 3 years preceding the first COVID-19-related imaging study, routine demographic data, biochemical and hematological data, and outcome data.

In the European Union (EU), SoBigData is a research initiative under the EU's Horizon 2020 programme (Grant No. 654024 and 871042) which provides an integrated ecosystem of “big data” for ethnic-sensitive scientific discoveries in multiple fields including mathematics, ICT, and human, social, and economic sciences (SoBigData.eu, 2021). The idea is to promote repeatable and open science by meeting the data and infrastructural needs of researchers while also ensuring that users' data are gathered for specific application and timebound (e.g., relates to dealing with the COVID-19 pandemic only and data will be deleted afterwards), the data cannot be shared without consent, and the data must be of direct benefit to the users whose data were gathered. Another initiative in the EU, The Confederation of Laboratories for Artificial Intelligence (CLAIRE) (CLAIRE, 2021), has warned that it is very likely that our societies will be confronted with other crises at a scale similar to COVID-19 in the not-so-far future and have outlined an European approach with the recommendation that standards and frameworks that would facilitate the development of efficient management of medical data that will not erode human dignity must be developed (Ishmaev et al., 2021).

In the United States, three national institutions namely, National Center for Advancing Translational Sciences (NCATS), Clinical and Translational Science Awards (CTSA) Program and Institutional Development Award Networks for Clinical and Translational Research (IDeA-CTR), have partnered to form the National COVID Cohort Collaborative (N3C) in an attempt to enable collaborators to contribute and use COVID-19 clinical data for scientific research that will have impact in the battle against the pandemic (NCATS-US, 2021). As at the time of writing this paper, the data of more than 950,000 COVID-19 positive patients are available from N3C for researchers to examine associations between COVID-19 patient outcomes and other determinants of health and, at least, 144 projects are already on-going for this purpose. Interestingly, in addition to patient data being de-identified for privacy reasons, this cloud-based data repository consists of synthetic (that is, computationally derived) data that statistically resemble original patient information but are not the actual data of the patients, adding another layer of privacy protection for patients.

The summary of the key messages and the main lessons learnt on the application of AI-based techniques and frameworks for the detection, diagnosis and epidemiological predictions, forecasting and social control of COVID-19 is as follows:

- We recommend that healthcare centers set up dedicated teams with expertise in medical data collection, privacy, access and sharing, and data governance frameworks and protocols for pandemics and other emergency times.
- External model evaluation is important to avoid the problems associated with model overfitting and biasness, such as arriving at clinically unusable solutions or introducing inequalities in health and healthcare. We recommend the establishment of independent units at national level or through international collaboration with the goal of assessing and validating AI applications developed for healthcare during pandemics before such applications are adopted and scaled up.
- The regulatory landscape (covering issues such as safety, effectiveness and benefit-versus-harm analysis) governing the development of AI tools in healthcare need to be accessible and understandable to AI experts. We recommend that professional bodies that will oversee certification programmes for AI experts working in healthcare be introduced to ensure that, through continuing professional development, these professionals adhere to common ethical standards and are aware of the current ethical and social issues related to their work.
- The COVID-19 pandemic has highlighted the importance of domain specific knowledge in AI, and multidisciplinary research collaborations will only accelerate the translation of AI research into practical and useful solutions in healthcare. In funding AI projects, we recommend that research fund awarding bodies should make the collaboration between AI scientists and domain specific experts a condition for grant awards.
- In order to gain the trust of the population in terms of data collection, privacy and protection, we recommend that all stakeholders work together in the development of a data use and sharing framework that will ensure effective data management is in place for the development and advancement of AI applications in healthcare.

## Conclusion

In this paper, AI techniques that are being used in the detection, diagnosis and epidemiological predictions, forecasting and social control for combating COVID-19 have been discussed. While AI has huge potential in the battle against COVID-19, the successful practical deployments of these AI-based tools have so far been limited due to challenges such as limited data accessibility, need for external evaluation of AI models, lack of awareness of AI experts of the regulatory landscape governing the deployment of AI tools in healthcare, the need for clinicians and other experts to work with AI experts in a multidisciplinary context and the need to address public concerns over data collection, privacy and protection. Overcoming these challenges will lead to significant progress in battling COVID-19 and future pandemics using AI techniques.

## Author Contributions

MA drafted the first version of the manuscript. MA and SP contributed to the content and writing of the final version. Both authors contributed to the article and approved the submitted version.

## Conflict of Interest

The authors declare that the research was conducted in the absence of any commercial or financial relationships that could be construed as a potential conflict of interest.
